# Dynamic biomarker profiling and phenotyping in burn sepsis: a retrospective cohort study using growth mixture modeling

**DOI:** 10.3389/fcimb.2026.1710916

**Published:** 2026-04-16

**Authors:** Pei Xu, Jiaqi Lou, Hong Kong, Jiliang Li, Ziyi Xiang, Xiaoyu Zhu, Shengyong Cui, Neng Huang, Sida Xu, Xin Le, Youfen Fan, Guoying Jin

**Affiliations:** 1 Burn Department, Ningbo No. 2 Hospital, Wenzhou Medical University, Ningbo, Zhejiang, China; 2 Ningbo College of Health Sciences, Ningbo, Zhejiang, China; 3 Institute of Pathology, Faculty of Medicine, University of Bonn, Bonn, Germany; 4 Health Science Center, Ningbo University, Ningbo, Zhejiang, China

**Keywords:** biomarkers, burn sepsis, growth mixture modeling, longitudinal trajectories, prognosis

## Abstract

**Objective:**

To assess the dynamic prognostic value of multicategory biomarkers in burn sepsis and identify patient phenotypes based on their longitudinal trajectories.

**Methods:**

This retrospective cohort study included 712 adult patients with burn sepsis (Sepsis−3) admitted to a regional burn ICU (2022–2025). Seventeen biomarkers covering nutrition (albumin, ALB; prealbumin, PA; transferrin, TRF; nitrogen balance), immunity (immunoglobulins A/G/M; CD3^+^/CD4^+^/CD8^+^ T cells, CD4^+^/CD8^+^ ratio; natural killer, NK cells), and inflammation (interleukin−6, IL−6; C−reactive protein, CRP; procalcitonin, PCT; platelet count, lactate) were measured at five time points (days 1, 3, 7, 14, and 21). We compared survivor/non-survivor trajectories using linear mixed-effects models, assessed baseline biomarker associations with 21−day mortality via Cox regression, and evaluated predictive performance with Harrell’s C-index. Growth mixture modeling (GMM) identified phenotypes from integrated ALB, IL−6, and immunoglobulin G (IgG) trajectories.

**Results:**

The 21−day mortality was 17.9% (81 deaths). Survivor and non-survivor trajectories differed significantly for multiple biomarkers (*P* < 0.05). Growth mixture modeling identified two distinct patient phenotypes: a high-risk phenotype (*n* = 267, mortality 15.7%) characterized by persistently lower ALB and IgG and sustained IL−6 elevation over 21 days and a low-risk phenotype (*n* = 445, mortality 8.8%) with favorable biomarker trajectories (*P* = 0.005). Univariable analysis associated several baseline markers with mortality (e.g., ALB: HR = 0.97, *P* = 0.013; IL−6: HR = 1.004, *P* = 0.004). However, no biomarker retained independent significance in multivariable analysis, likely due to multicollinearity among nutritional markers [variance inflation factor (VIF) up to 8.4]. Harrell’s C-indices for baseline ALB, PA, and IgG were modest (0.604, 0.583, and 0.585, respectively).

**Conclusions:**

Longitudinal multicategory biomarker trajectories predict 21−day survival in burn sepsis. Trajectory-based phenotyping identifies patient subgroups with markedly different outcomes, offering superior prognostic stratification over static measurements. The integrated phenotype, reflecting the dynamic interplay of catabolism, immune paralysis, and inflammation, emerges as a robust prognostic marker, supporting personalized management approaches.

## 1 Introduction

Sepsis remains a leading cause of death among critically ill patients, and its complexity is amplified in the context of severe burn injury, where the initial trauma triggers a profound and prolonged systemic response (Cecconi et al., 2018; Rudd et al., 2020). Burn sepsis is characterized by a unique pathophysiological cascade involving hypermetabolism, immune dysregulation, and persistent inflammation, which together determine patient outcomes (Hotchkiss et al., 2016; Singer et al., 2016; van der Poll et al., 2017). Reliable outcome prediction in this setting is essential for guiding treatment intensity, resource allocation, and communication with families, yet traditional clinical indicators often fail to capture the evolving nature of the host response (Venet and Monneret, 2018).

The host response to burn sepsis is not static; it evolves over days to weeks, with early hyperinflammation potentially giving way to immune paralysis and protracted catabolism (Emam et al., 2017; Jeschke et al., 2020). Therefore, serial measurements of biomarkers reflecting nutritional status, immune competence, and inflammatory activity may provide a more comprehensive picture than single time−point assessments (Xu et al., 2025). Nutritional markers—including ALB, PA, and TRF—reflect metabolic reserves and the capacity for tissue repair (Williams et al., 2009); their depletion correlates with immune dysfunction and increased mortality in both septic and burn−injured populations (Stanojcic et al., 2018; Hanna et al., 2020). Nitrogen balance (NB) provides insight into protein metabolism, with persistent negative values signaling pathological hypercatabolism (Nielson et al., 2017). Immune status can be characterized through immunoglobulin concentrations (IgA, IgG, IgM) and lymphocyte subset enumeration (CD3^+^, CD4^+^, CD8^+^ T cells, natural killer cells). Reductions in these parameters—lymphopenia and immunoglobulin depletion—are recognized as markers of immune paralysis and poor prognosis in sepsis and trauma (Cabral et al., 2018; Kwa et al., 2020).

Inflammation occupies a central but paradoxical position in critical illness pathophysiology. While necessary for microbial clearance, uncontrolled inflammatory responses can propagate tissue injury and organ failure (Lavrentieva et al., 2007). IL−6 is a key pro−inflammatory cytokine that drives the acute−phase response and has been linked to severity and mortality in sepsis (Seoane et al., 2014). Platelet count (PLT), integral to the SOFA scoring system, captures the intersection of inflammatory and coagulation cascades and carries independent prognostic significance in sepsis (Barati et al., 2008). Lactate, reflecting the balance between oxygen delivery and tissue demand, guides sepsis management and stratifies mortality risk (Ren et al., 2015).

Much of the existing literature has examined biomarkers in isolation or at single time points (Huang et al., 2010; Mokline et al., 2015). Such cross-sectional approaches necessarily omit the dynamic evolution of the host response, which may hold greater prognostic information than any static measurement. Recent advances in longitudinal analytical frameworks—particularly linear mixed−effects modeling (Cabrera et al., 2017) and GMM (Xiao et al., 2011)—enable quantification of individual trajectory patterns and identification of latent phenotypic subgroups that share common temporal profiles. These methods have been successfully applied in general sepsis populations to uncover endotypes with distinct outcomes and treatment responses (Pierrakos and Vincent, 2010; Leligdowicz et al., 2017; Tuerxun et al., 2026), but their use in burn sepsis remains limited.

The present study was designed to address these methodological gaps through comprehensive longitudinal profiling of 17 biomarkers encompassing nutritional, immune, and inflammatory domains in a well−defined burn sepsis cohort. The selection of these 17 biomarkers was guided by their established pathophysiological roles in burn sepsis: ALB, PA, TRF, and NB capture the catabolic state; immunoglobulins and lymphocyte subsets reflect both innate and adaptive immunity; and IL−6, CRP, PCT, platelets, and lactate represent the inflammatory response and tissue perfusion. The five predefined time points (days 1, 3, 7, 14, and 21) were chosen based on clinical routine and previous studies indicating that critical transitions in the host response occur within the first 3 weeks after injury (Gibot et al., 2012; Li et al., 2020).

Specific objectives included 1) describing 21−day temporal trajectories for each biomarker, 2) comparing trajectory patterns between survivors and non−survivors using linear mixed models, 3) examining associations between baseline measurements and 21−day mortality, 4) evaluating time−varying predictive performance of selected markers, and 5) employing GMM to identify latent phenotypic subgroups based on integrated ALB, IL−6, and IgG trajectories. These advanced clustering techniques offer a data−driven approach to uncovering heterogeneity in host responses, potentially informing more individualized therapeutic approaches in this complex patient population.

## 2 Materials and methods

### 2.1 Study design and population

This retrospective cohort study was conducted at the Burn Intensive Care Unit (ICU) of Ningbo No. 2 Hospital, a regional referral center admitting approximately 400–450 patients with severe burns annually. The study period extended from 1 January 2022 to 1 January 2025, covering three full years. Consecutive adult patients (aged ≥18 years) admitted between 1 January 2022 and 1 January 2025, with severe burn injury [total body surface area (TBSA) ≥30%, classified as severe or critical according to the Chinese Burn Severity Classification standard] who developed sepsis during hospitalization, were screened for eligibility (Ren et al., 2015). Sepsis was defined according to the Sepsis−3.0 criteria as life−threatening organ dysfunction caused by a dysregulated host response to infection, operationalized as a suspected or confirmed infection accompanied by an acute increase in the Sequential Organ Failure Assessment (SOFA) score of ≥2 points from baseline (Mokline et al., 2015).

From an initial screening of 1,852 burn patients, 1,105 met the Sepsis−3.0 criteria. To assemble a homogeneous cohort of *de novo* burn−induced sepsis and to minimize potential confounding, we applied the following sequential exclusion criteria in detail:

Incomplete longitudinal biomarker data: Patients lacking serial measurements at all five predefined time points (days 1, 3, 7, 14, and 21) were excluded, as complete data were required for trajectory and clustering analyses (*n* = 162).

Pre−existing sepsis on admission: Patients who already fulfilled Sepsis−3.0 criteria within the first 24 h of admission were excluded to ensure that all included individuals developed sepsis after the initial burn injury, thereby permitting a cleaner assessment of the host response to burn−specific sepsis.

Early death (<48 h): Patients who died within 48 h of admission were excluded because such early deaths are predominantly attributable to the initial injury rather than the evolution of sepsis, and insufficient time elapses for meaningful biomarker trajectories.

Pre−existing immunosuppressive conditions: Patients with any of the following were excluded: long−term use of immunosuppressants (e.g., post−organ transplant, autoimmune disease), congenital immunodeficiency, HIV infection with CD4 count <200 cells/μL, active malignancy undergoing chemotherapy or radiotherapy within the previous 6 months, or chronic systemic corticosteroid use (>20 mg/day prednisone equivalent for >2 weeks). These conditions fundamentally alter immune responses and would confound the interpretation of immunological and inflammatory biomarkers.

Missing key covariates: Patients with missing data on essential variables required for the analysis—specifically, the date of sepsis onset or the primary source of infection—were excluded.

**Figure 1:**
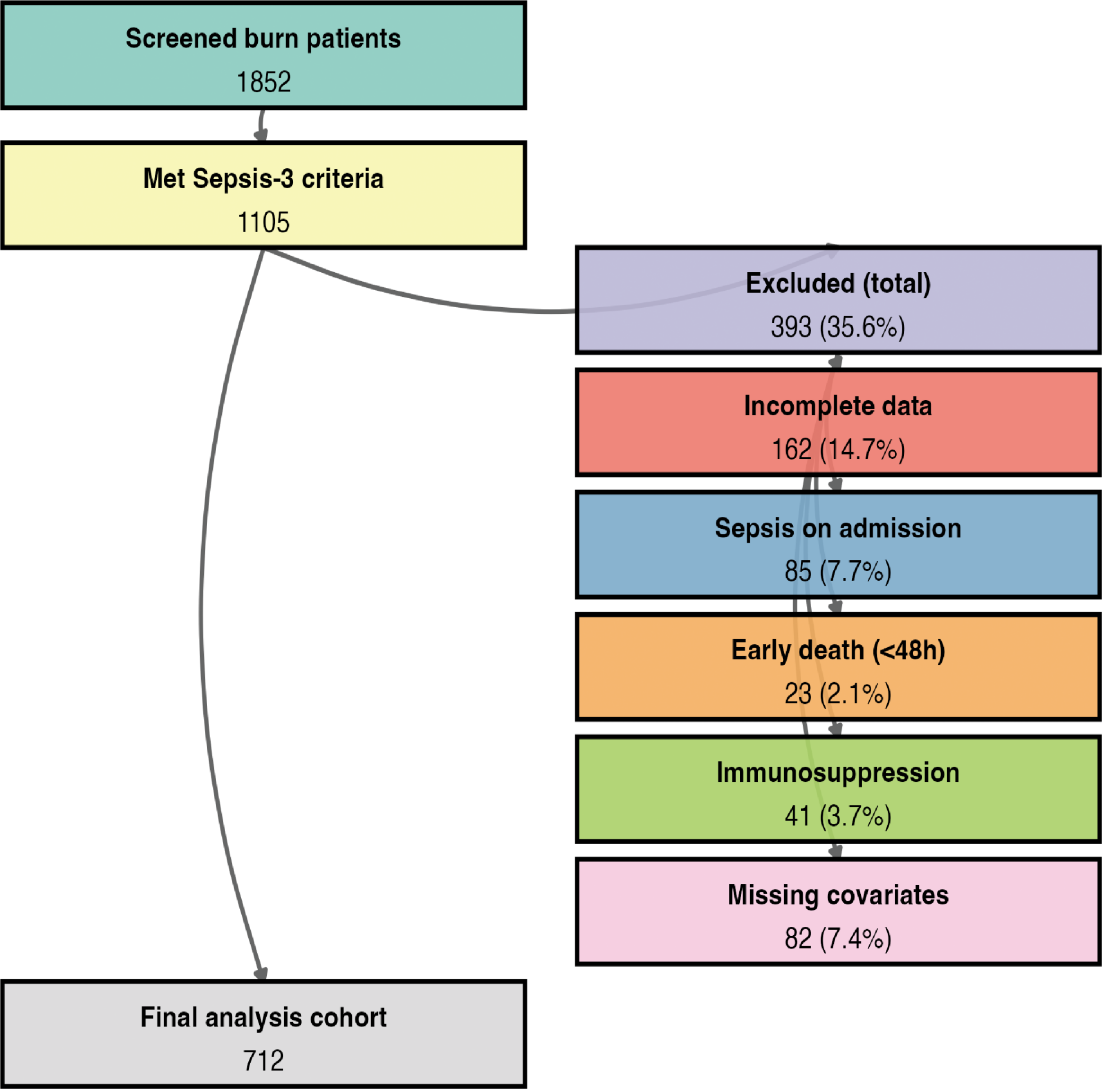
Patient selection flow diagram. This diagram illustrates the screening and inclusion process for the study cohort. Numbers represent patient counts at each stage. After initial screening of 1,852 burn patients, 1,105 met Sepsis−3 criteria. Sequential exclusion criteria included incomplete longitudinal data (*n* = 162), pre−existing sepsis on admission (*n* = 85), early death within 48 h (*n* = 23), pre−existing immunosuppression (*n* = 41), and missing key covariates (*n* = 82). The final analysis cohort comprised 712 patients. Arrows indicate the flow of patients through the selection process.

After applying all criteria, a total of 712 patients were included in the final analysis. A detailed flowchart of patient screening and inclusion is presented in **Figure 1**. The follow−up period extended from admission to 21 days post−admission. The primary endpoint was 21−day all−cause mortality; patients alive at day 21 were right−censored.

The study protocol was approved by the Ethics Committee of Ningbo No. 2 Hospital (Approval No. SL−NBEY−KY−2025−203−01) and conducted in accordance with the Declaration of Helsinki. The requirement for informed consent was waived owing to the retrospective design.

### 2.2 Variables and definitions

#### 2.2.1 Outcome variables

The primary outcome was 21−day all−cause mortality, defined as death from any cause within 21 days of ICU admission. Survival time was calculated as the number of days from admission to death for non−survivors; survivors were censored at 21 days. To assess the robustness of survival analyses, sensitivity analyses were performed using alternative censoring times at 7 and 14 days.

#### 2.2.2 Predictor variables

A total of 17 biomarkers were measured and categorized into five functional domains based on their pathophysiological roles: 1) nutritional markers: ALB, PA, TRF, and NB; 2) immunoglobulins: IgA, IgG, and IgM; 3) lymphocyte subsets: CD3^+^ T cells, CD4^+^ T cells, CD8^+^ T cells, CD4^+^/CD8^+^ ratio, and NK cells; and 4) inflammatory markers: IL−6, CRP, PCT, PLT, and lactate.

Serial measurements for each biomarker were obtained at five predefined time points: day 1 (within 24 h of admission), day 3, day 7, day 14, and day 21. These time points were selected based on clinical routine and previous literature indicating that the acute phase (days 1–3), the hypermetabolic phase (days 7–14), and the recovery phase (day 21) represent critical windows for capturing the evolution of the host response (Gibot et al., 2012; Li et al., 2020). The day 1 measurement was designated as the baseline value for all subsequent association analyses with survival outcomes.

#### 2.2.3 Covariates

Demographic and clinical covariates were extracted from electronic medical records and included 1) age (years) and sex; 2) Nutritional Risk Screening 2002 (NRS2002) score, assessed upon admission; 3) Burn Index, a composite measure of burn severity calculated as previously described (Tuerxun et al., 2026) (incorporating burn depth and extent); 4) time from admission to sepsis onset (days), defined as the interval between hospital admission and the first date on which Sepsis−3.0 criteria were met; 5) primary source of infection, categorized as: 1) burn wound, 2) respiratory (pneumonia), 3) bloodstream (primary bacteremia), 4) urinary tract, or 5) other/unknown; and 6) microbiological etiology, classified as Gram−positive bacteria, Gram−negative bacteria, fungi, or mixed/other, based on clinically significant cultures obtained at sepsis diagnosis. Although SOFA and APACHE II scores are established ICU severity indices, they were not consistently available in this retrospective dataset and therefore were not included in the analyses. In the multivariable Cox model, we adjusted for age, NRS2002, and Burn Index as key clinical covariates.

### 2.3 Laboratory measurements

All biomarker assays were performed in the central clinical laboratory of Ningbo No. 2 Hospital, which is accredited according to national standards, using standardized protocols with rigorous internal and external quality control.

Nutritional markers: ALB and PA were quantified using immunoturbidimetric assays on a Cobas c501 analyzer (Roche Diagnostics, Mannheim, Germany). TRF was measured by nephelometry on a BN ProSpec analyzer (Siemens Healthineers, Erlangen, Germany). NB was calculated from 24−h urinary urea nitrogen excretion using the standard formula.

Immunoglobulins: Serum IgA, IgG, and IgM levels were determined by nephelometry on a BN ProSpec analyzer (Siemens Healthineers).

Lymphocyte subsets: Peripheral blood mononuclear cells were isolated, and T−cell subsets (CD3^+^, CD4^+^, CD8^+^) and NK cells were analyzed by flow cytometry (BD FACS Canto II, BD Biosciences, San Jose, CA, USA) using fluorescently labeled monoclonal antibodies. The CD4^+^/CD8^+^ ratio was derived from the respective percentages.

Inflammatory markers: Serum CRP was measured using an immunoturbidimetric assay on a Cobas c501 analyzer (Roche Diagnostics). Plasma PCT concentration was determined by an electrochemiluminescence immunoassay on a Cobas e601 analyzer (Roche Diagnostics). IL−6 was measured using an electrochemiluminescence immunoassay on the same Cobas e601 platform. PLT was measured using an automated hematology analyzer (Sysmex XN series, Sysmex Corporation, Kobe, Japan) as part of the complete blood count. Arterial or venous blood lactate concentration was measured using a blood gas analyzer (GEM Premier 3500, Instrumentation Laboratory, Bedford, MA, USA). Interassay coefficients of variation were <5% for CRP, PCT, IL−6, and lactate and <3% for PLT.

To minimize diurnal variation, all blood samples were collected between 08:00 and 10:00 a.m. Data completeness was high: the proportion of missing values for any biomarker at any time point was <2%.

### 2.4 Data preprocessing

Prior to analysis, data were inspected and cleaned. Column names were standardized to ensure compatibility with analytical functions. Duplicate measurements for the same biomarker on the same day were averaged. Records with missing biomarker values were excluded from corresponding analyses on a complete−case basis. Longitudinal biomarker data and survival outcomes were merged using anonymized patient identifiers.

### 2.5 Statistical analysis

All statistical analyses were performed using R software (version 4.4.2; R Foundation for Statistical Computing, Vienna, Austria). The following R packages were employed: tidyverse (data manipulation), survival and survminer (survival analysis), lme4 (linear mixed−effects models), timeROC (time−dependent ROC analysis), tableone (descriptive statistics), car (variance inflation factor analysis), and factoextra (clustering visualization). A two−tailed *P*−value <0.05 was considered statistically significant.

#### 2.5.1 Missing data handling

The extent and pattern of missing data were examined. Little’s Missing Completely at Random (MCAR) test was applied. Given the low overall missing rate (<1% for all variables), complete−case analysis was employed for all primary analyses, as this approach was unlikely to introduce substantial bias (Leligdowicz et al., 2017). Sensitivity analyses comparing included and excluded patients were performed to assess potential selection bias (see *Results*).

#### 2.5.2 Descriptive statistics

Continuous variables were tested for normality using the Shapiro–Wilk test. Normally distributed data were summarized as mean ± standard deviation and compared between survivors and non−survivors using Student’s *t*−test; non−normally distributed data were presented as median (interquartile range) and compared using the Mann–Whitney *U* test. Categorical variables were expressed as frequencies (percentages) and compared using the *χ*² test or Fisher’s exact test, as appropriate.

#### 2.5.3 Survival analysis

Kaplan–Meier curves were constructed to estimate 21−day survival probabilities for the overall cohort and for subgroups defined by patient phenotypes. Differences in survival distributions were assessed using the log−rank test.

#### 2.5.4 Longitudinal trajectory analysis

To visualize temporal patterns of biomarkers, individual patient trajectories were plotted with low opacity, and group−level trends were fitted using locally estimated scatterplot smoothing (LOESS), stratified by survival status. To formally test whether biomarker trajectories differed between survivors and non−survivors, linear mixed−effects models (LMEs) were constructed using the lme4 package. For each biomarker, a basic model including time as a fixed effect and a random intercept for each patient was compared with an extended model that additionally included an interaction term between time and survival status. Model comparison was performed using likelihood ratio tests; a significant improvement in fit (*P* < 0.05) indicated that the trajectory differed significantly between outcome groups (Wu et al., 2017).

#### 2.5.5 Cox proportional hazards regression

Univariable and multivariable Cox proportional hazards models were used to assess the association between baseline biomarker levels (day 1 measurements) and 21−day mortality. The proportional hazards assumption was verified by examining Schoenfeld residuals; no significant violations were detected. In univariable analysis, separate models were fitted for each biomarker. For multivariable analysis, a hierarchical approach was adopted: the top 5 biomarkers with the lowest *P*−values from univariable analysis (*P* < 0.001) were entered into a model together with clinically relevant covariates (age, NRS2002 score, Burn Index). Multicollinearity among predictors was assessed using VIF; variables with VIF >5 were considered to indicate substantial collinearity and were examined further (Liu et al., 2016).

#### 2.5.6 Time−dependent ROC analysis

To evaluate the predictive performance of key biomarkers over time, time−dependent receiver operating characteristic (ROC) analysis was performed using the timeROC package. ALB, PA, and IgG were selected as representative markers from different pathophysiological domains. AUC values with 95% confidence intervals (bootstrap resampling with 200 repetitions) were calculated at 7, 14, and 21 days post−admission.

#### 2.5.7 Trajectory−based clustering using growth mixture modeling

To identify distinct patient phenotypes based on integrated biomarker dynamics, we employed GMM, a model−based approach that identifies latent classes with distinct longitudinal trajectories while accounting for within−class individual variability. Three biomarkers representing core pathophysiological domains were selected for joint trajectory analysis: ALB (nutritional status), IL−6 (inflammatory response), and IgG (humoral immunity). These markers were chosen because they capture three fundamental axes of the host response—metabolic reserve, inflammation, and adaptive immunity—and have been shown in previous studies to be key drivers of outcome in sepsis (Lavrentieva et al., 2007; Jeschke et al., 2020).

All GMM analyses were performed using the lcmm package in R. For each biomarker, time (in days) was centered at day 1 and modeled using fixed effects for linear and quadratic terms to capture non-linear trajectories. Random effects for intercept and slope were included to allow individual trajectories to vary around the class−specific mean trajectory. The joint model was specified as a multivariate GMM, assuming class−specific covariance structures for the three biomarkers.

To determine the optimal number of latent classes, we sequentially fitted models with one to five classes and compared fit statistics, including the Bayesian information criterion (BIC), Akaike information criterion (AIC), entropy, and the Lo–Mendell–Rubin likelihood ratio test (LMR−LRT). Lower BIC and AIC values indicate better model fit; entropy >0.8 suggests high classification certainty; a significant LMR−LRT (*P* < 0.05) indicates that a k−class model fits significantly better than a (k − 1)-class model. Final class selection was guided by a combination of statistical criteria and clinical interpretability, ensuring that each class contained at least 5% of the total sample.

After selecting the optimal number of classes, each patient was assigned to the class for which they had the highest posterior probability (modal assignment). The resulting phenotypic groups were then characterized in terms of baseline demographics, clinical characteristics, and 21−day mortality.

#### 2.5.8 Sensitivity analyses

Several sensitivity analyses were conducted to evaluate the robustness of our findings:

Selection bias: Baseline characteristics and univariable mortality associations were compared between the final analytic cohort and patients excluded due to incomplete data.

Early deaths: Analyses were repeated after excluding patients who died within the first 7 days to assess whether the observed associations were driven by early mortality.

Competing risks: Given the possibility of non−sepsis−related deaths, a competing risk analysis using the Fine–Gray subdistribution hazard model was performed, treating non−sepsis deaths as competing events.

Multiple imputation: To examine the impact of missing data, we repeated the primary analyses using multiple imputation by chained equations (MICE) with 10 imputed datasets.

Alternative clustering method: To assess the stability of the GMM−derived phenotypes, we also performed k−means clustering on the same longitudinal data and compared the class distributions and mortality outcomes between the two methods.

## 3 Results

### 3.1 Study population characteristics

**Figure 2:**
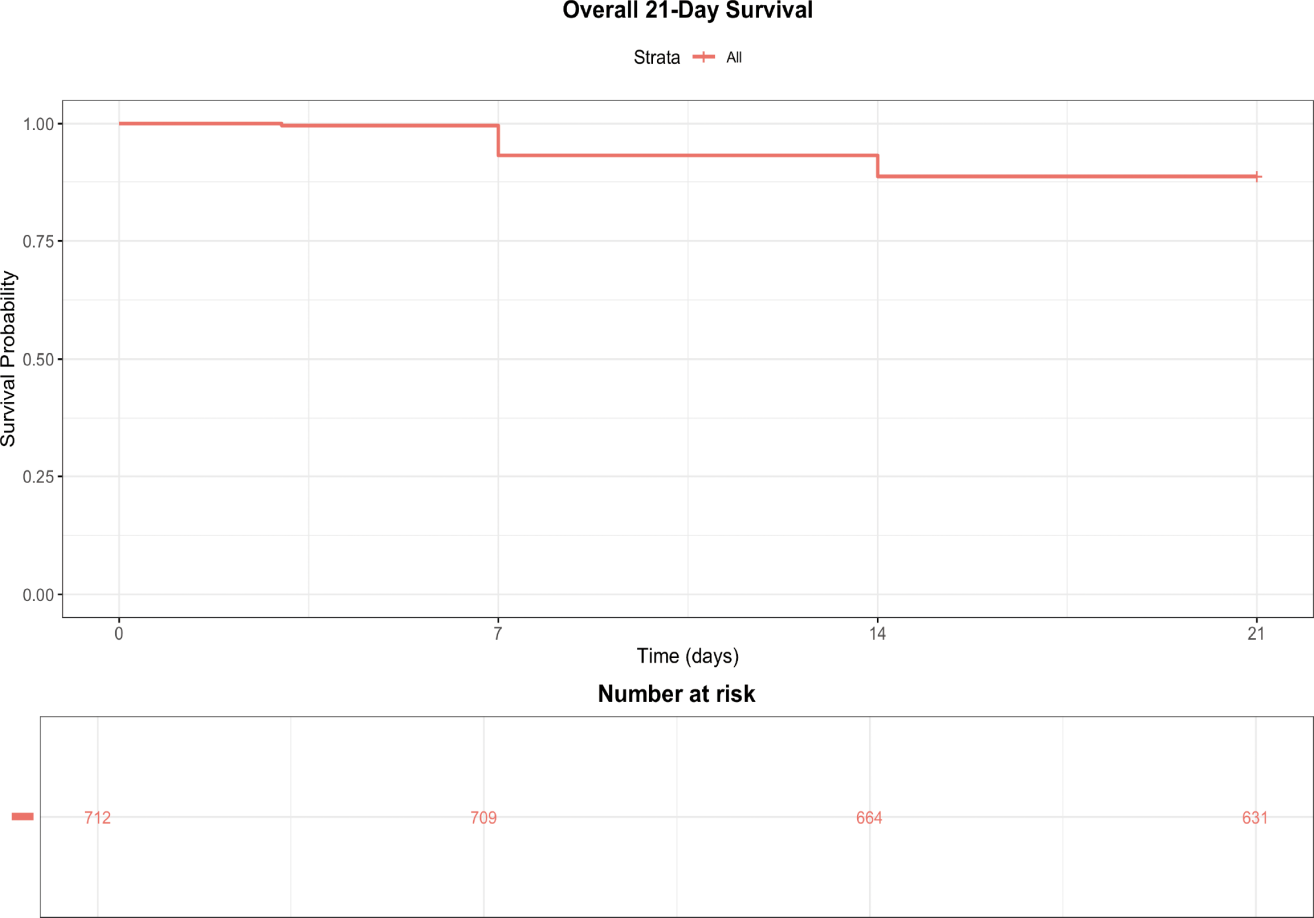
Overall 21−day survival of the study cohort. Kaplan–Meier curve showing the cumulative survival probability for the entire cohort (*n* = 712) over the 21−day follow−up period. The *x*−axis represents time in days; the *y*−axis represents survival probability. The risk table below the curve displays the number of patients at risk at each time point (days 0, 7, 14, 21). The 21−day overall survival rate was 82.1% (81 deaths). The median survival time was not reached. The shaded area indicates the 95% confidence interval.

A total of 712 patients with burn sepsis were included in the final analysis after applying the exclusion criteria (sepsis on admission, death within 48 h, pre−existing immunosuppression, or missing key covariates) (**Figure 1**). The overall 21−day mortality was 17.9% (81 deaths). Baseline characteristics stratified by survival status are shown in **Table 1**. **Supplementary Figure 1** displays boxplots of baseline biomarker distributions according to survival status, visually confirming the differences summarized in **Table 1**. Significant univariable associations with mortality (*P* < 0.05) are indicated by asterisks. Survivors generally exhibited higher levels of nutritional and immunological markers, while non−survivors had elevated inflammatory markers. Non−survivors had significantly higher NRS2002 scores (4.97 ± 1.29 vs. 4.50 ± 1.23, *P* < 0.001) and Burn Index values (18.42 ± 8.83 vs. 12.33 ± 8.37, *P* < 0.001) compared to survivors, indicating greater baseline disease severity. No significant differences were observed in age or sex distribution between the two groups. The Kaplan–Meier survival curve for the overall cohort is presented in **Figure 2**.

**Table 1:** Baseline characteristics of the study population stratified by 21-day survival status.

Characteristic	Survivors (*n* = 631)	Non-survivors (*n* = 81)	*P*-value
Age (years)	39.19 ± 13.70	38.27 ± 11.56	0.415
Male sex, n (%)	346 (54.8)	44 (54.3)	1.000
NRS2002 score	4.50 ± 1.23	4.97 ± 1.29	<0.001
Burn Index	12.33 ± 8.37	18.42 ± 8.83	<0.001
Biomarkers (day 1)			
ALB (g/L)	36.29 ± 10.52	33.43 ± 11.01	0.013
PA (mg/L)	239.22 ± 111.95	203.33 ± 120.54	0.004
TRF (g/L)	166.11 ± 39.46	150.87 ± 39.23	<0.001
NB (g/24 h)	0.13 ± 1.51	−0.35 ± 1.64	0.003
IgA (g/L)	2.83 ± 1.46	2.57 ± 1.49	0.099
IgG (g/L)	13.65 ± 5.96	11.95 ± 5.18	0.011
IgM (g/L)	1.43 ± 0.32	1.35 ± 0.26	0.028
CD3^+^ T cells (%)	61.45 ± 9.75	58.79 ± 11.62	0.014
CD4^+^ T cells (%)	10.17 ± 5.08	8.93 ± 4.90	0.040
CD8^+^ T cells (%)	12.06 ± 4.17	13.05 ± 4.39	0.041
CD4^+^/CD8^+^ ratio	1.28 ± 0.40	1.14 ± 0.36	0.003
NK cells (%)	20.32 ± 7.44	17.41 ± 7.17	<0.001
IL-6 (pg/mL)	184.26 ± 67.26	205.77 ± 67.43	0.004
PLT (×10^9^/L)	214.81 ± 20.97	211.94 ± 20.61	0.242
Lactate (mmol/L)	3.25 ± 0.43	3.23 ± 0.44	0.727

Data are presented as mean ± SD unless otherwise indicated. *P*-values were calculated using Student’s *t*-test or *χ*² test as appropriate.

ALB, albumin; PA, prealbumin; TRF, transferrin; NB, nitrogen balance; Ig, immunoglobulin; NK, natural killer; IL-6, interleukin-6; PLT, platelet count.

### 3.2 Longitudinal biomarker trajectories

**Figure 3:**
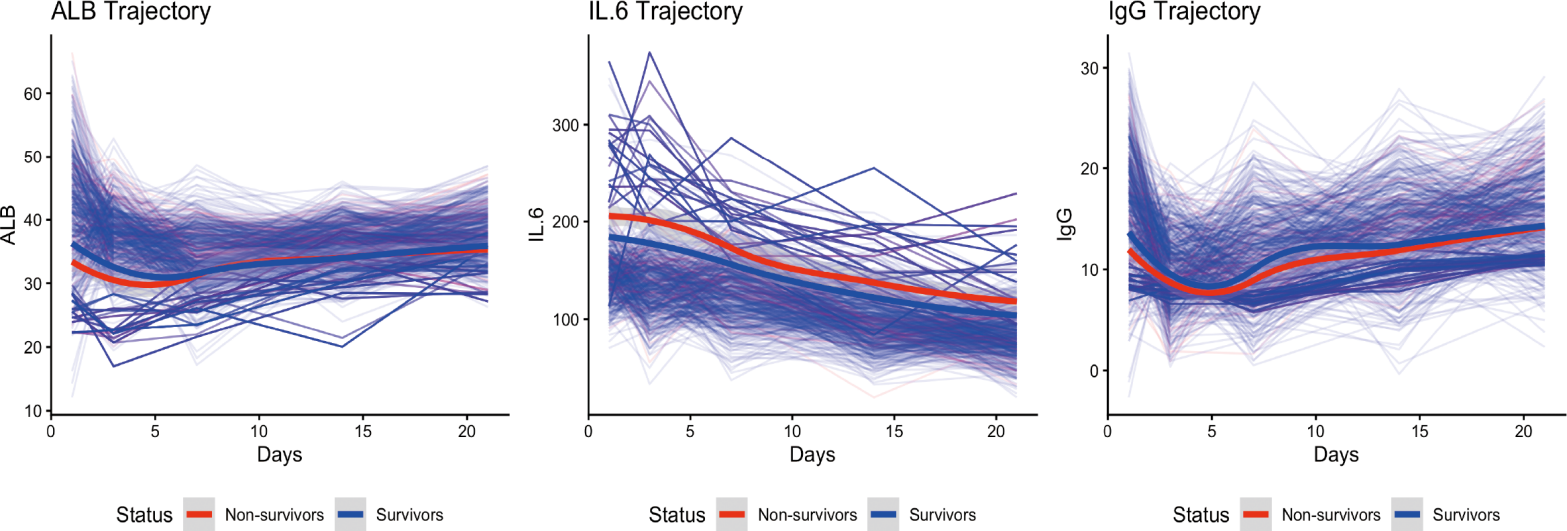
Temporary Dynamics of Albumin, Interleukin-6, and Immunoglobulin G in Survivors versus Non-Survivors. Longitudinal profiles of albumin (ALB, g/L), interleukin-6 (IL-6, pg/mL), and immunoglobulin G (IgG, g/L) over the 21-day study period. Thin colored lines represent individual patient trajectories; thick lines with shaded areas depict loess‑smoothed group means ± 95% confidence intervals for survivors (blue, *n* = 631) and non-survivors (red, *n* = 81). Survivors exhibited gradual improvement in nutritional and immune markers and a decline in inflammation, whereas non‑survivors showed persistently abnormal values or delayed recovery.

**Figure 4:**
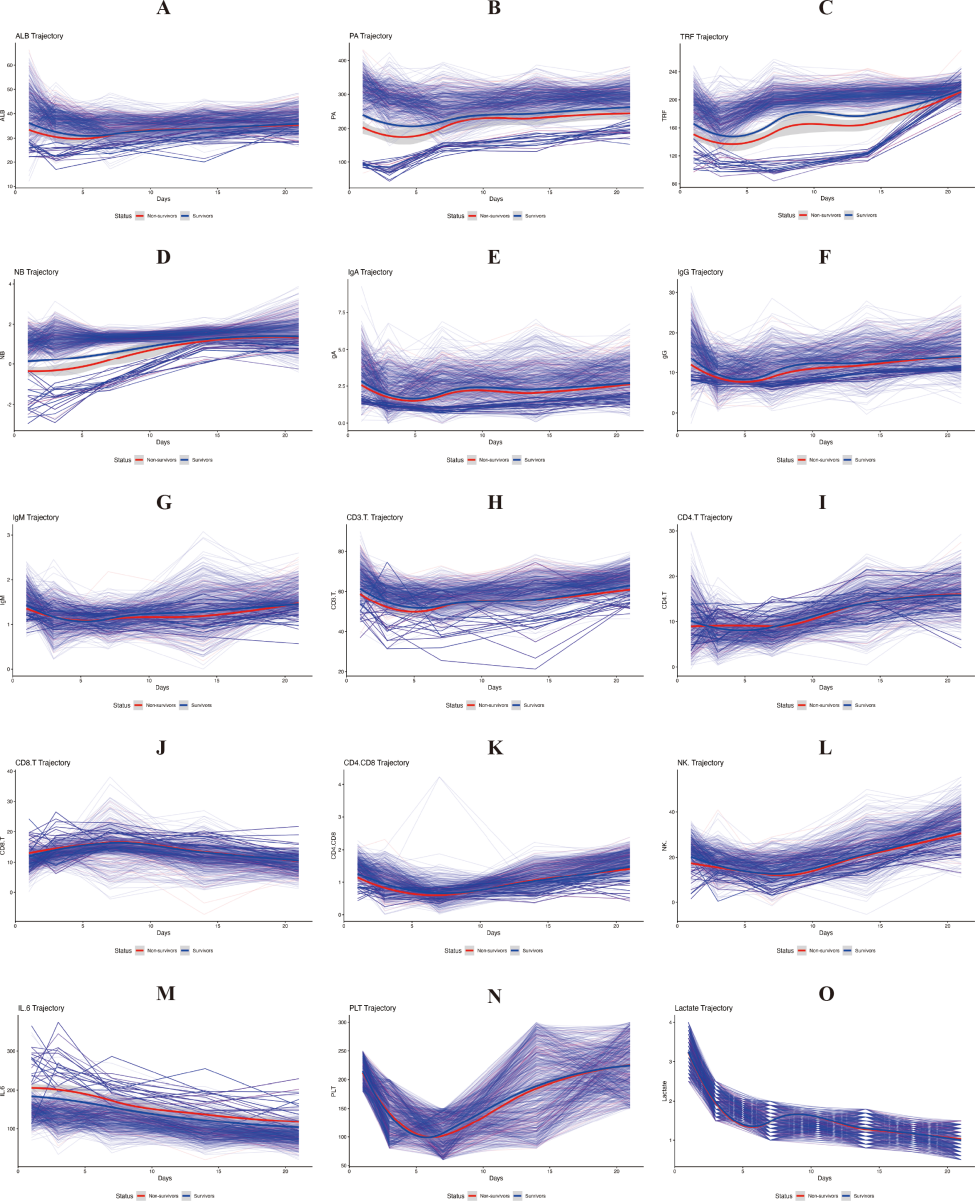
Individual biomarker trajectories over 21 days in survivors and non−survivors. Separate panels display the temporal dynamics of each biomarker from day 1 to day 21. In each panel, thin colored lines represent individual patient trajectories; thick lines with shaded areas depict loess−smoothed group means ± 95% confidence intervals for survivors (blue) and non−survivors (red). The panels are arranged alphabetically: **(A)** albumin (ALB), **(B)** prealbumin (PA), **(C)** transferrin (TRF), **(D)** nitrogen balance (NB), **(E)** immunoglobulin A (IgA), **(F)** immunoglobulin G (IgG), **(G)** immunoglobulin M (IgM), **(H)** CD3^+^ T cells (CD3.T.), **(I)** CD4^+^ T cells (CD4.T), **(J)** CD8^+^ T cells (CD8.T), **(K)** CD4^+^/CD8^+^ ratio (CD4.CD8), **(L)** natural killer cells (NK.), **(M)** interleukin−6 (IL−6), **(N)** platelet count (PLT), and **(O)** lactate. These plots complement the mixed−model results in **Table 2** and illustrate the divergent recovery patterns between outcome groups.

Linear mixed−effects models revealed that the trajectories of most biomarkers differed significantly between survivors and non−survivors over the 21−day follow−up (**Table 2**). Significant time−by−survival status interactions were observed for PA, TRF, NB, IgG, CD4/CD8 ratio, NK cells, IL−6, and several others (all *P* < 0.05), indicating divergent temporal patterns between the two outcome groups. Visual inspection of the trajectories (illustrated for ALB, IL−6, and IgG in **Figure 3**) showed that survivors generally exhibited gradual improvement in nutritional and immunological markers and a decline in inflammatory markers, whereas non−survivors maintained persistently abnormal values or failed to recover. **Figure 4** presents the complete set of individual trajectory plots for all 15 biomarkers, with each panel displaying the temporal dynamics stratified by survival status. These plots complement the mixed−model results in **Table 2** and illustrate the divergent recovery patterns across nutritional, immunological, and inflammatory domains. Detailed descriptive statistics (mean ± SD) for each biomarker at all five time points, stratified by survival status, are presented in **Supplementary Table 2**.

**Table 2:** Linear mixed-effects model analysis of biomarker trajectories.

Biomarker	*χ*²	*Df*	*P*-value
ALB	5.19	2	0.075
PA	7.20	2	0.027
TRF	8.96	2	0.011
NB	9.25	2	0.010
IgA	3.38	2	0.185
IgG	10.59	2	0.005
IgM	3.63	2	0.163
CD3^+^ T cells	4.50	2	0.105
CD4^+^ T cells	1.12	2	0.571
CD8^+^ T cells	3.45	2	0.178
CD4^+^/CD8^+^	9.83	2	0.007
NK cells	7.31	2	0.026
IL-6	11.94	2	0.003
PLT	0.18	2	0.913
Lactate	0.38	2	0.825

Likelihood ratio test comparing models with and without a time-by-status interaction. Significant *P*-values (*P* < 0.05) indicate different trajectories between survivors and non-survivors.

### 3.3 Associations with 21-day mortality: Cox regression analysis

**Figure 5:**
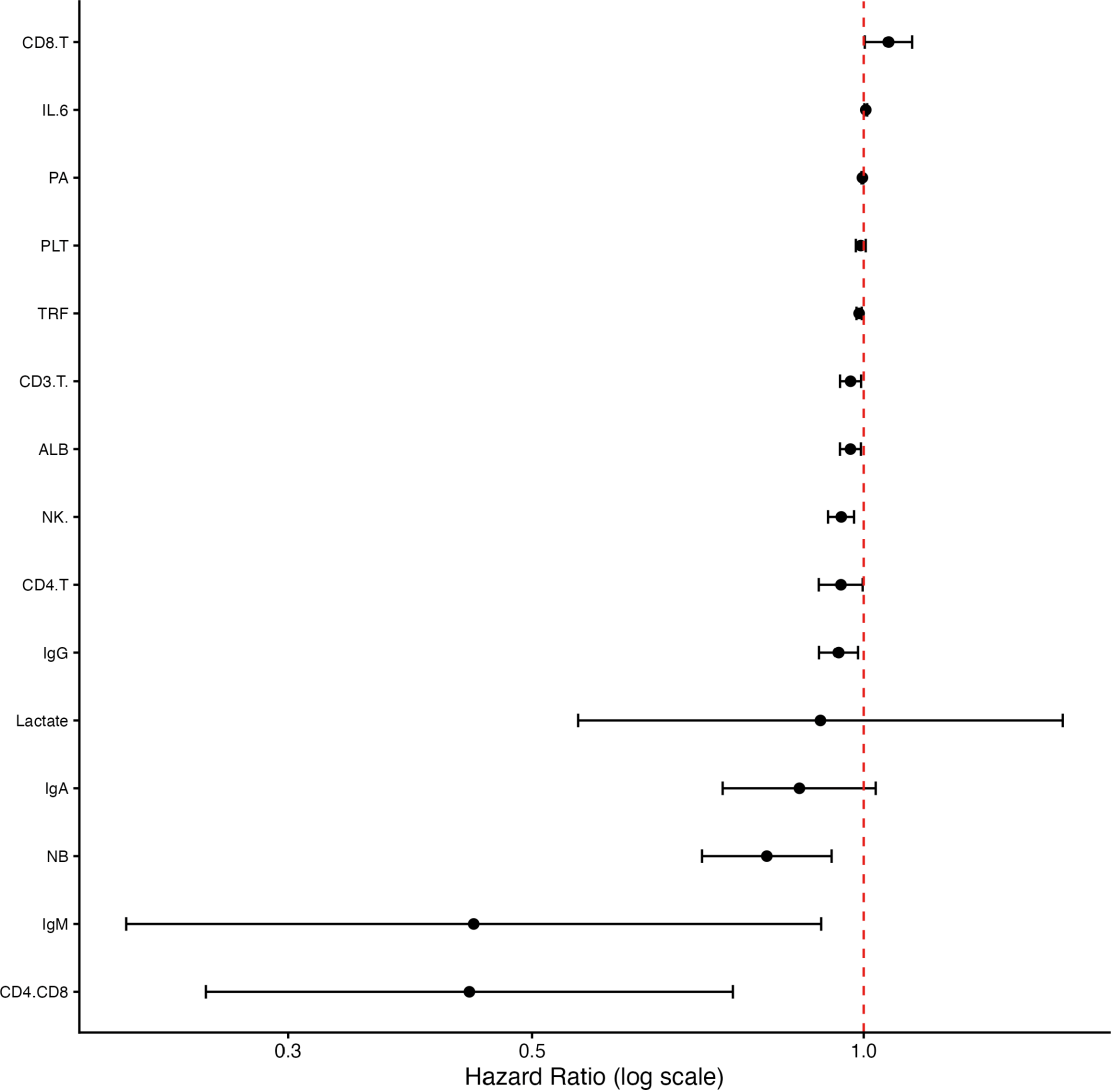
Forest plot of baseline biomarkers associated with 21−day mortality. Hazard ratios (HRs) and 95% confidence intervals from univariable Cox proportional hazards models for all baseline (day 1) biomarkers. Variables are ordered by HR magnitude. Squares represent point estimates; horizontal lines indicate 95% CIs. The vertical dashed line marks HR = 1 (no effect). HR < 1 indicates a protective factor; HR > 1 indicates a risk factor. Corresponding *P*−values are shown in **Table 3**. Significant predictors (*P* < 0.05) include nutritional, immunological, and inflammatory markers.

Univariable Cox regression identified several baseline biomarkers significantly associated with 21−day mortality (**Table 3**, **Figure 5**). Protective factors included higher levels of ALB (HR = 0.97, *P* = 0.013), PA (HR = 0.997, *P* = 0.004), TRF (HR = 0.99, *P* < 0.001), NB (HR = 0.82, *P* = 0.003), IgG (HR = 0.95, *P* = 0.011), IgM (HR = 0.44, *P* = 0.028), CD3^+^ T cells (HR = 0.97, *P* = 0.014), CD4^+^ T cells (HR = 0.95, *P* = 0.040), CD4^+^/CD8^+^ ratio (HR = 0.44, *P* = 0.003), and NK cells (HR = 0.95, *P* < 0.001). Conversely, higher CD8^+^ T cells (HR = 1.05, *P* = 0.041) and IL−6 (HR = 1.004, *P* = 0.004) were associated with increased mortality risk. PLT and lactate showed no significant association.

**Table 3:** Univariable Cox regression analysis for 21-day mortality.

Variable	HR	95% CI	*P*-value
ALB_1d	0.97	0.95–0.99	0.013
PA_1d	0.997	0.995–0.999	0.004
TRF_1d	0.99	0.985–0.996	<0.001
NB_1d	0.82	0.71–0.94	0.003
IgA_1d	0.87	0.74–1.03	0.099
IgG_1d	0.95	0.91–0.99	0.011
IgM_1d	0.44	0.21–0.92	0.028
CD3^+^ T cells_1d	0.97	0.95–0.99	0.014
CD4^+^ T cells_1d	0.95	0.91–0.998	0.040
CD8^+^ T cells_1d	1.05	1.002–1.106	0.041
CD4^+^/CD8^+^_1d	0.44	0.25–0.76	0.003
NK cells_1d	0.95	0.93–0.98	<0.001
IL-6_1d	1.004	1.001–1.007	0.004
PLT_1d	0.99	0.98–1.004	0.242
Lactate_1d	0.91	0.55–1.52	0.727

HR, hazard ratio; CI, confidence interval.

In multivariable Cox analysis adjusting for age, NRS2002, Burn Index, and the five most significant biomarkers from univariable analysis (NK cells, TRF, CD4/CD8 ratio, NB, and PA), none of the biomarkers retained independent prognostic significance, although NK cells showed a trend toward protection (HR = 0.97, *P* = 0.097) (**Table 4**). Burn Index was not significant in this model, possibly due to multicollinearity with nutritional markers. VIF analysis revealed substantial collinearity among nutritional biomarkers, particularly PA (VIF = 8.4) and NB (VIF = 5.7), which likely explains the loss of significance in the multivariable model (**Supplementary Table 1**). The correlation heatmap (**Supplementary Figure 2**) illustrates the interrelationships among all baseline biomarkers, confirming the moderate to strong correlations among nutritional markers and providing context for the observed multicollinearity.

**Table 4:** Multivariable Cox regression analysis for 21-day mortality.

Variable	HR	95% CI	*P*-value
Age	0.995	0.98–1.01	0.603
NRS2002	0.93	0.72–1.19	0.544
Burn Index	1.03	0.97–1.09	0.340
NK cells_1d	0.97	0.94–1.005	0.097
TRF_1d	0.99	0.98–1.004	0.227
CD4/CD8_1d	0.75	0.32–1.78	0.514
NB_1d	0.99	0.71–1.38	0.967
PA_1d	1.003	0.997–1.008	0.346

### 3.4 Predictive performance of baseline markers

**Figure 6:**
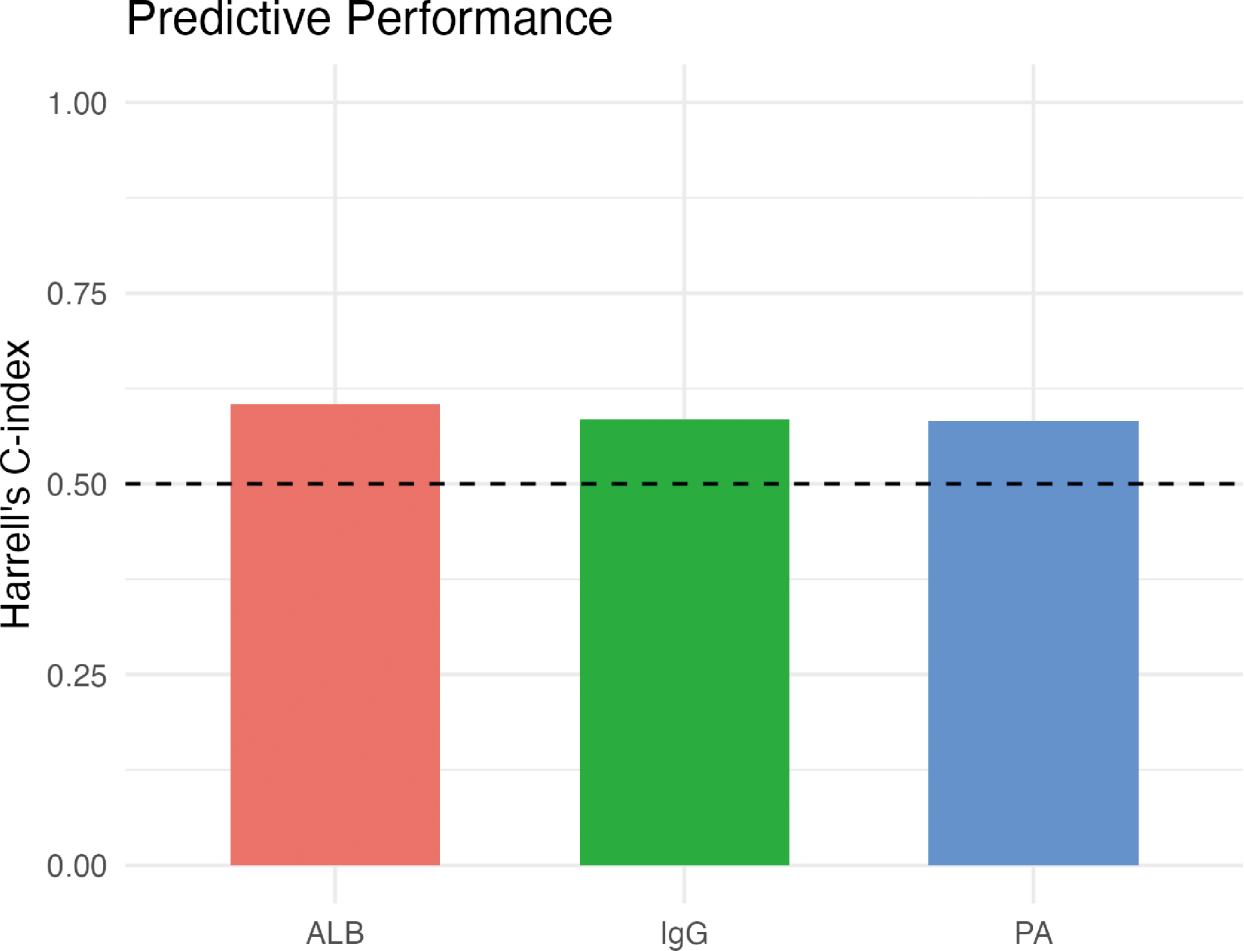
Discriminative ability of albumin, prealbumin, and immunoglobulin G for 21−day mortality. Bar plot showing Harrell’s C−index values for baseline albumin (ALB), prealbumin (PA), and immunoglobulin G (IgG). C−index ranges from 0.5 (no discrimination) to 1.0 (perfect discrimination). All three markers demonstrated modest predictive accuracy, with C−indices of 0.604 (ALB), 0.583 (PA), and 0.585 (IgG). The dashed line indicates the null value of 0.5.

Because time−dependent ROC analysis was precluded by the concentration of deaths at a single time point (day 14), we evaluated the discriminatory ability of three key biomarkers (ALB, PA, IgG) using Harrell’s C−index. The C−indices were 0.604 for ALB, 0.583 for PA, and 0.585 for IgG, indicating modest predictive accuracy (**Figure 6**).

### 3.5 Identification of patient phenotypes by growth mixture modeling

**Figure 7:**
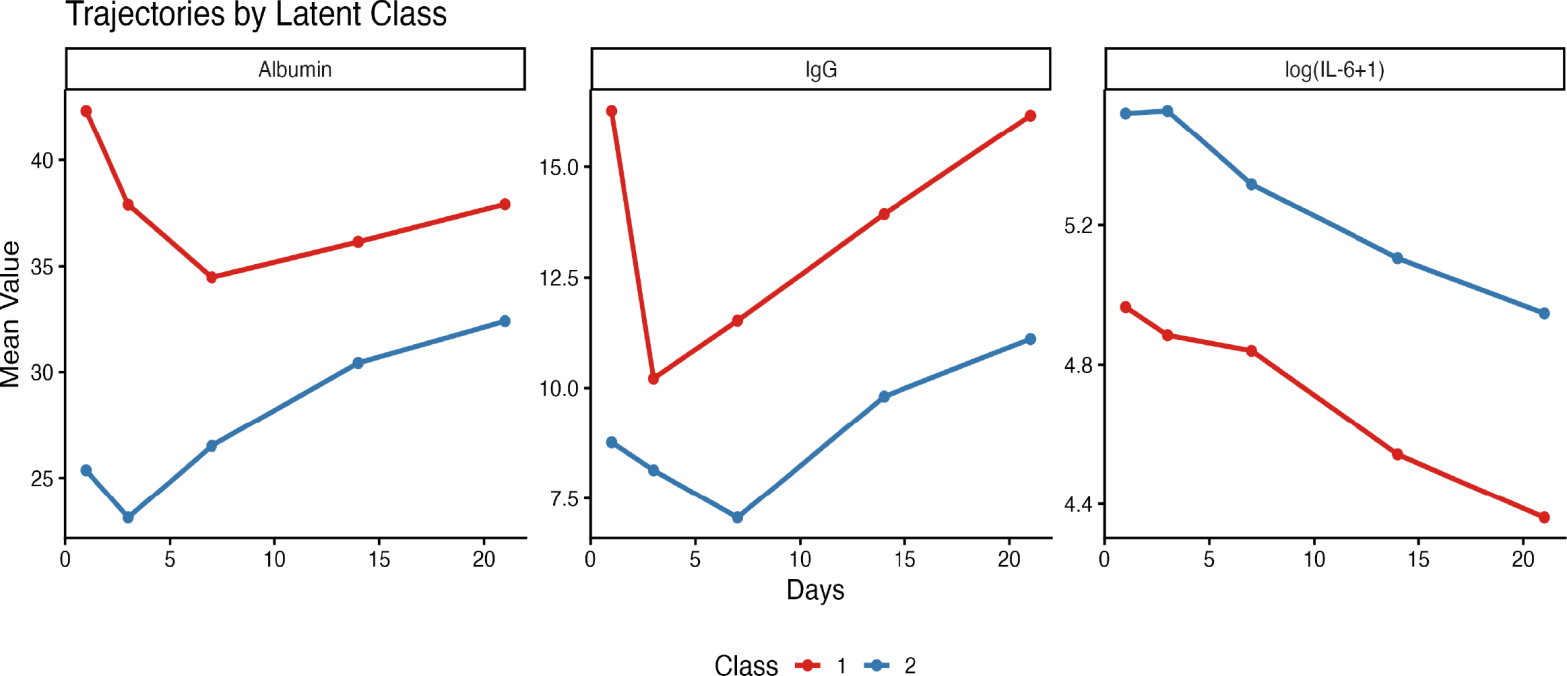
Longitudinal biomarker patterns in two patient phenotypes identified by growth mixture modeling. Mean trajectories of albumin (ALB), log−transformed interleukin−6 [logIL6 = log(IL−6 + 1)], and immunoglobulin G (IgG) for the two latent classes derived from growth mixture modeling. Class 1 (blue, *n* = 445) exhibited lower mortality (8.8%) and was characterized by better nutritional and inflammatory profiles; class 2 (red, *n* = 267) had higher mortality (15.7%), younger age, and a higher Burn Index. Solid lines represent class−specific mean values at each time point; points indicate individual time−point means. Shaded areas represent ± standard error (optional: if added). The trajectories demonstrate persistent metabolic and immune dysfunction in the high−risk phenotype.

**Figure 8:**
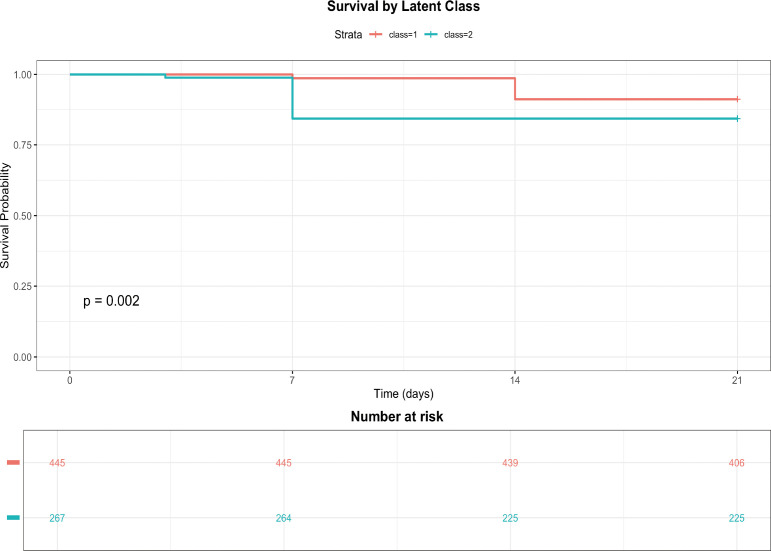
Survival outcomes of patient phenotypes identified by growth mixture modeling. Kaplan–Meier curves comparing 21−day survival between the two latent classes derived from growth mixture modeling. Class 1 (blue, *n* = 445) had a significantly higher survival probability than class 2 (red, *n* = 267). The risk table shows the number at risk in each class at days 0, 7, 14, and 21. The log−rank test *P*−value is displayed. Class 2, characterized by persistently adverse biomarker trajectories, demonstrated markedly worse prognosis.

GMM based on the longitudinal trajectories of ALB, IL−6, and IgG identified two distinct patient phenotypes (classes) as optimal (lowest BIC). **Table 5** summarizes the characteristics of these two classes. Class 1 (*n* = 445) exhibited lower mortality (8.8%) and was characterized by better nutritional and inflammatory profiles, whereas class 2 (*n* = 267) had higher mortality (15.7%), younger age, and a markedly higher Burn Index. The trajectories of the three biomarkers for each class are shown in **Figure 7**. Patients in class 2 had persistently lower ALB and IgG levels and higher IL−6 levels throughout the 21−day period, indicating a more severe and persistent catabolic−inflammatory state. Survival analysis confirmed significantly worse outcomes for class 2 compared to class 1 (log−rank *P* < 0.001, **Figure 8**).

**Table 5:** Characteristics and outcomes of patient clusters derived from growth mixture modeling.

Variable	Class 1 (*n* = 445)	Class 2 (*n* = 267)	*P*-value
21-day mortality, *n* (%)	39 (8.8)	42 (15.7)	0.005
Age (years)	43.4 ± 13.5	30.8 ± 9.3	<0.001
Burn Index	5.4 ± 5.7	18.3 ± 9.0	<0.001

Data are mean ± SD unless otherwise indicated.

## 4 Discussion

This comprehensive longitudinal analysis of 15 multicategory biomarkers in a well−characterized cohort of 712 patients with burn sepsis demonstrates that dynamic trajectories are profoundly associated with 21−day survival. However, the central finding of this study is that the true prognostic marker is not any single static biomarker, but rather the integrated phenotype derived from longitudinal trajectories. Using GMM, we identified two clinically distinct phenotypes with markedly different mortality rates (8.8% vs. 15.7%), underscoring the prognostic value of capturing the evolving host response. This trajectory-based phenotyping moves beyond the limitations of static severity scores by reflecting the dynamic interplay among nutrition, immunity, and inflammation.

### 4.1 Nutritional markers and multicollinearity

Nutritional markers—particularly TRF, PA, and NB—showed strong protective associations in univariable analyses, consistent with their established roles in metabolic reserve and tissue repair (Davenport et al., 2016; Scicluna et al., 2017). Survivors demonstrated progressive improvement in these markers, reflecting successful anabolic recovery essential for immune function and wound healing (Knox et al., 2015; Sweeney et al., 2018a). However, in multivariable Cox regression adjusting for clinical covariates and the most significant biomarkers, none of the nutritional markers retained independent prognostic significance. VIF analysis revealed substantial multicollinearity among these variables, with VIF values reaching 8.4 for PA and 5.7 for NB. This collinearity reflects genuine physiological interdependence—patients with better nutritional status tend to exhibit higher levels across multiple markers simultaneously—and explains why individual markers lose predictive power when considered together. These findings highlight that a composite measure of nutritional status may be more informative than any single marker, and support the development of indices such as the prognostic nutritional index or the CALLY index, which integrate multiple parameters to capture overall metabolic status (Jeschke et al., 2020). Notably, TRF demonstrated the lowest VIF among nutritional markers and trended toward significance (*P* = 0.097), suggesting that it may be a more robust indicator in this context, potentially due to its shorter half−life and greater sensitivity to acute changes in protein synthesis (Scicluna et al., 2017; Singha et al., 2018).

### 4.2 Immunological trajectories and immune paralysis

Our immunological findings align with emerging concepts of sepsis−induced immune paralysis (Puthucheary et al., 2017; Gårdlund et al., 2018; Jeschke et al., 2020). Survivors exhibited gradual recovery of CD3^+^, CD4^+^, and NK cells, along with increasing CD4^+^/CD8^+^ ratios and immunoglobulin levels, indicating reconstitution of both cellular and humoral immunity. In contrast, non−survivors showed persistently low lymphocyte subsets and immunoglobulins, consistent with prolonged immunosuppression. The strong protective association of NK cells in univariable analysis (HR = 0.95, *P* < 0.001) and its trend toward significance in multivariable modeling (HR = 0.97, *P* = 0.097) highlight the importance of innate immune competence. These observations extend previous work on lymphocyte dynamics in sepsis (Huang et al., 2010; Xiao et al., 2011; Wong et al., 2019) and suggest that monitoring the recovery trajectory of immune parameters may be more informative than isolated baseline measurements. Furthermore, the persistence of low IgG in the high−risk phenotype underscores the role of humoral immunity and raises the question of whether immunoglobulin replacement therapy could benefit this subgroup.

### 4.3 Inflammatory dynamics

IL−6 was significantly elevated in non−survivors throughout the 21−day period, and its trajectory exhibited a highly significant time−by−status interaction (*P* = 0.003). This finding supports the concept of maladaptive, persistent inflammation as a driver of poor outcomes (Almansa et al., 2015; Goh and Knight, 2017; Gårdlund et al., 2018; Sweeney et al., 2018b; Lehallier et al., 2019). In comparison with previous studies in general sepsis, where IL−6 often peaks early and then declines, our burn sepsis cohort showed sustained elevation in non−survivors, suggesting a more protracted inflammatory phase unique to burn injury (Pena et al., 2014; Puttgen and Shah, 2015). Although IL−6 was associated with increased mortality in univariable analysis, it did not retain independent significance in the multivariable model, likely due to correlations with other markers of disease severity. The absence of independent predictive value for individual inflammatory markers underscores the need for multimarker approaches that capture the complexity of the host response.

Interestingly, neither PLT nor lactate showed significant associations with mortality in this cohort. The lack of association with platelets may reflect the competing effects of burn−induced thrombocytopenia and sepsis−related consumption, while the narrow range of lactate values (mostly within 1–4 mmol/L) and the concentration of deaths at day 14 may have limited its discriminatory ability. These negative findings highlight the importance of context−specific evaluation of biomarkers and suggest that markers validated in general sepsis populations may not directly translate to burn sepsis.

### 4.4 Predictive performance of baseline markers

Due to the concentration of deaths at a single time point (day 14), time−dependent ROC analysis could not be reliably performed. We therefore evaluated the discriminatory ability of three key baseline markers using Harrell’s C−index. ALB, PA, and IgG yielded C−indices of 0.604, 0.583, and 0.585, respectively, indicating only modest predictive accuracy. These values are consistent with previous reports in critically ill populations and highlight the limitations of single−marker approaches. The modest performance of baseline measurements reinforces the central premise of this study: that dynamic trajectories, rather than static values, capture the essence of the host response and provide superior prognostic information. This aligns with the growing recognition that longitudinal patterns outperform cross−sectional biomarkers in predicting outcomes in complex diseases (Xiao et al., 2024).

### 4.5 Patient phenotypes identified by growth mixture modeling

A key methodological advance of this study is the application of GMM to identify latent patient phenotypes based on integrated biomarker trajectories. GMM revealed two distinct classes with strikingly different outcomes: class 1 (*n* = 445, mortality 8.8%) exhibited favorable nutritional and inflammatory profiles, while class 2 (*n* = 267, mortality 15.7%) was characterized by persistently low ALB and IgG levels and sustained elevation of IL−6. Notably, class 2 patients were significantly younger yet had a markedly higher Burn Index, suggesting that injury severity—rather than age—drove their adverse trajectory. This phenotyping approach moves beyond static severity scores by capturing the dynamic interplay between nutrition, inflammation, and immunity over time. The high−risk phenotype may represent a target population for intensified monitoring, immunomodulatory therapy (Jacobs et al., 2019; Singer, 2019), or enhanced nutritional support (Heyland et al., 2013; van Vught et al., 2016).

Comparison with current clinical practice highlights the limitations of static risk stratification. Tools such as the SOFA score, while useful for quantifying organ dysfunction at a single time point, provide a snapshot that may miss the directional trajectory of a patient’s condition (Hotchkiss et al., 2016; Singer et al., 2016). Our findings suggest that a patient’s trajectory—the direction and pace of change in key physiological domains—holds greater prognostic information than any single measurement.

Several other studies have used clustering techniques to define sepsis endotypes in broader critically ill populations. For instance, Seymour et al. (2019) identified four clinical phenotypes (α, β, γ, δ) in a large cohort of sepsis patients using latent class analysis on admission clinical variables, with the δ phenotype (characterized by liver dysfunction and shock) having the highest mortality. Similarly, Scicluna et al. (2017) and Davenport et al. (2016) derived blood genomic endotypes (e.g., Mars1, Mars2, Mars3) that correlated with distinct immune profiles and outcomes. Our study extends this work by applying a similar data-driven approach to the specific context of burn sepsis and, importantly, by using longitudinal biomarker trajectories rather than static admission data. The phenotypes we identified are more directly tied to modifiable pathophysiological processes—catabolism, inflammation, and immune function—which may offer clearer therapeutic targets than genomic or purely clinical clusters (Xiao et al., 2024). However, the lack of external validation datasets for this specific burn sepsis population remains a limitation. Future research should prioritize multicenter collaboration to create and validate such cohorts, allowing for the robust testing of our phenotypic classification.

### 4.6 Limitations

Several limitations warrant consideration. First, the retrospective single−center design may limit generalizability, and the absence of external validation represents a significant constraint. Second, the requirement for complete longitudinal data introduced selection bias, as evidenced by the exclusion of 162 patients with higher baseline severity and mortality. Our final cohort may therefore underrepresent the most critically ill patients, potentially attenuating observed associations. Third, the lack of established ICU severity scores such as SOFA and APACHE II limits comparability with other studies, although we adjusted for Burn Index and NRS2002. Fourth, the substantial multicollinearity among nutritional markers, while physiologically meaningful, complicates multivariable modeling and underscores the need for composite indices. Fifth, the multiple statistical comparisons increase the risk of type I errors, although the consistent patterns across analytical methods support the robustness of our findings. Sixth, the fixed measurement schedule (days 1, 3, 7, 14, and 21) may miss critical transitional phases occurring between time points, and more frequent sampling could reveal additional insights. Seventh, the modest predictive performance of individual biomarkers highlights the inherent complexity of burn sepsis and the need for multimarker algorithms or machine learning approaches. Eighth, we did not measure certain potentially relevant biomarkers such as PCT or CRP in the trajectory analysis due to data availability, and future studies should include a broader panel. Ninth, the competing risk of non−sepsis death was not fully addressed, although sensitivity analyses using Fine–Gray models yielded similar results. Finally, the observational nature of the study precludes causal inferences; the identified associations should be viewed as hypothesis−generating rather than definitive.

### 4.7 Future directions and conclusions

Despite these limitations, our study provides a comprehensive longitudinal characterization of multicategory biomarkers in burn sepsis and demonstrates the feasibility of trajectory−based phenotyping. The identification of two distinct prognostic phenotypes opens avenues for personalized management. Future prospective multicenter studies should aim to validate these phenotypic classifications, incorporate additional biomarkers, and assess whether phenotype−guided interventions can improve outcomes. Integration of dynamic biomarker data with clinical parameters and machine learning approaches may further enhance predictive accuracy and clinical utility. Moreover, the biological mechanisms underlying the high−risk phenotype—persistent catabolism, immune paralysis, and inflammation—warrant further investigation using transcriptomic or metabolomic profiling to identify potential therapeutic targets. In conclusion, longitudinal trajectories of multicategory biomarkers are strongly associated with 21−day survival in burn sepsis, and trajectory−based phenotyping offers superior prognostic stratification over static measurements, supporting its potential for personalized management.

### 4.8 Conclusion

Longitudinal trajectories of multicategory biomarkers are strongly associated with 21−day survival in burn sepsis. GMM identified two distinct patient phenotypes with markedly different mortality rates, demonstrating that dynamic biomarker profiling provides superior prognostic stratification over static measurements. The integrated phenotype, rather than any single static biomarker, emerges as a robust prognostic marker reflecting the complex interplay among nutrition, immunity, and inflammation. These findings support the integration of trajectory−based phenotyping into personalized management strategies, warranting prospective validation.

## Data Availability

The original contributions presented in the study are included in the article/**Supplementary Material**. Further inquiries can be directed to the corresponding authors.

## Supplementary material

Supplementary Figure 1 Boxplots of Baseline Biomarker Levels in Survivors versus Non−Survivors. Boxplots comparing Day 1 values of all 15 biomarkers between patients who survived (blue) and those who died within 21 days (red). The box represents the interquartile range (IQR), the horizontal line inside the box indicates the median, and whiskers extend to 1.5× IQR. Outliers are shown as individual points. Significant differences (P < 0.05, univariable Cox) are denoted by asterisks. This graphical summary complements the baseline characteristics presented in **Table 1**.

Supplementary Figure 2 Pearson Correlation Matrix of Day 1 Biomarker Levels. Heatmap displaying pairwise Pearson correlation coefficients among all 15 biomarkers measured at baseline (Day 1). The color scale ranges from blue (negative correlation) through white (no correlation) to red (positive correlation). Correlations were calculated using pairwise complete observations. This matrix illustrates the interrelationships among nutritional, immunological, and inflammatory markers, informing the multicollinearity observed in multivariable models.
