# Supplementary figures and images for: Dynamic biomarker profiling and phenotyping in burn sepsis: a retrospective cohort study using growth mixture modeling
Source: Front Cell Infect Microbiol. 2026 Apr 16;16:1710916. doi: 10.3389/fcimb.2026.1710916 (PMC13128616; doi:10.3389/fcimb.2026.1710916)

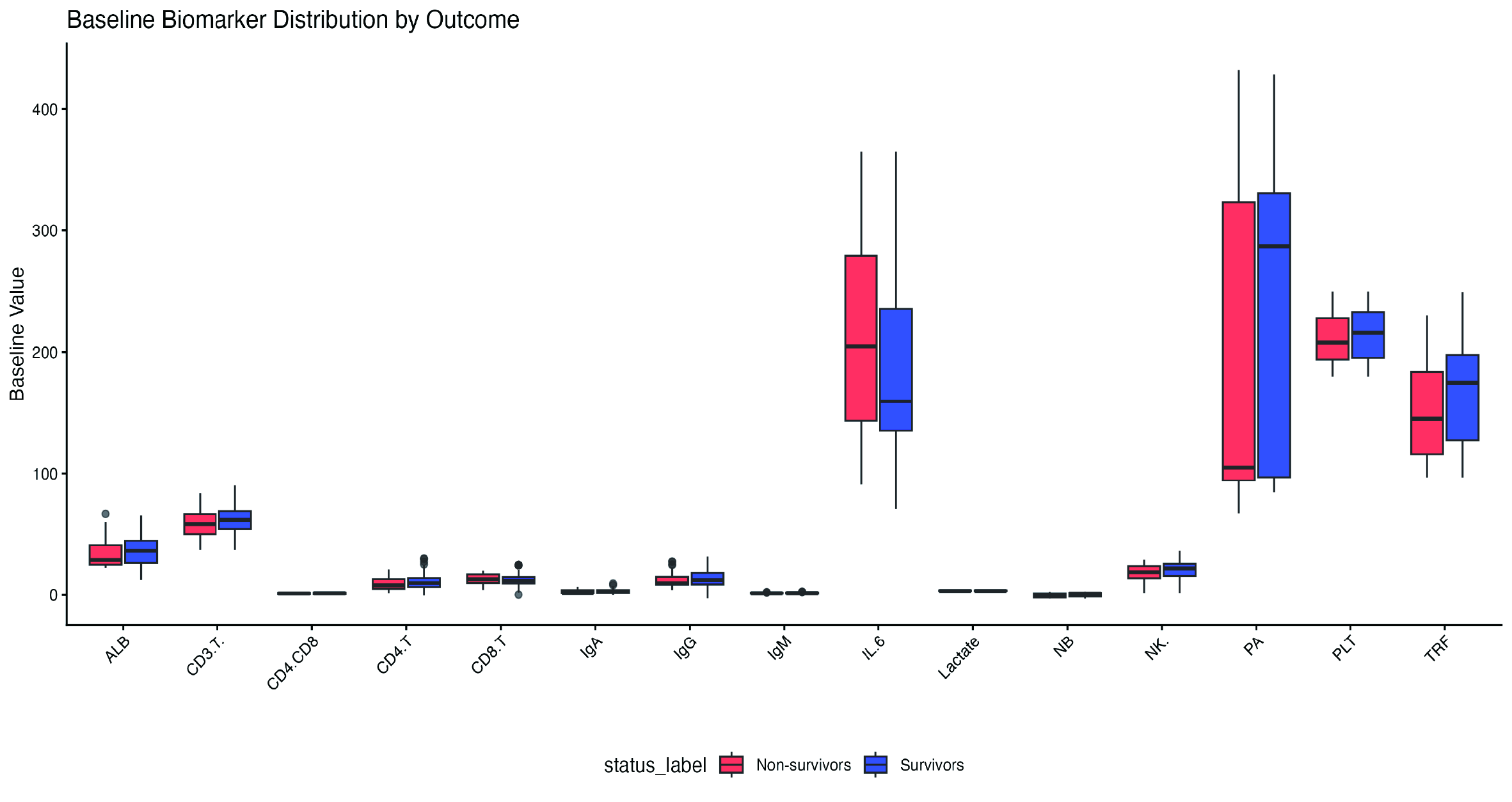

Supplement: Supplementary Figure 1 — Boxplots of Baseline Biomarker Levels in Survivors versus Non−Survivors. Boxplots comparing Day 1 values of all 15 biomarkers between patients who survived (blue) and those who died within 21 days (red). The box represents the interquartile range (IQR), the horizontal line inside the box indicates the median, and whiskers extend to 1.5× IQR. Outliers are shown as individual points. Significant differences (P < 0.05, univariable Cox) are denoted by asterisks. This graphical summary complements the baseline characteristics presented in Table 1. [file Image1.tif]

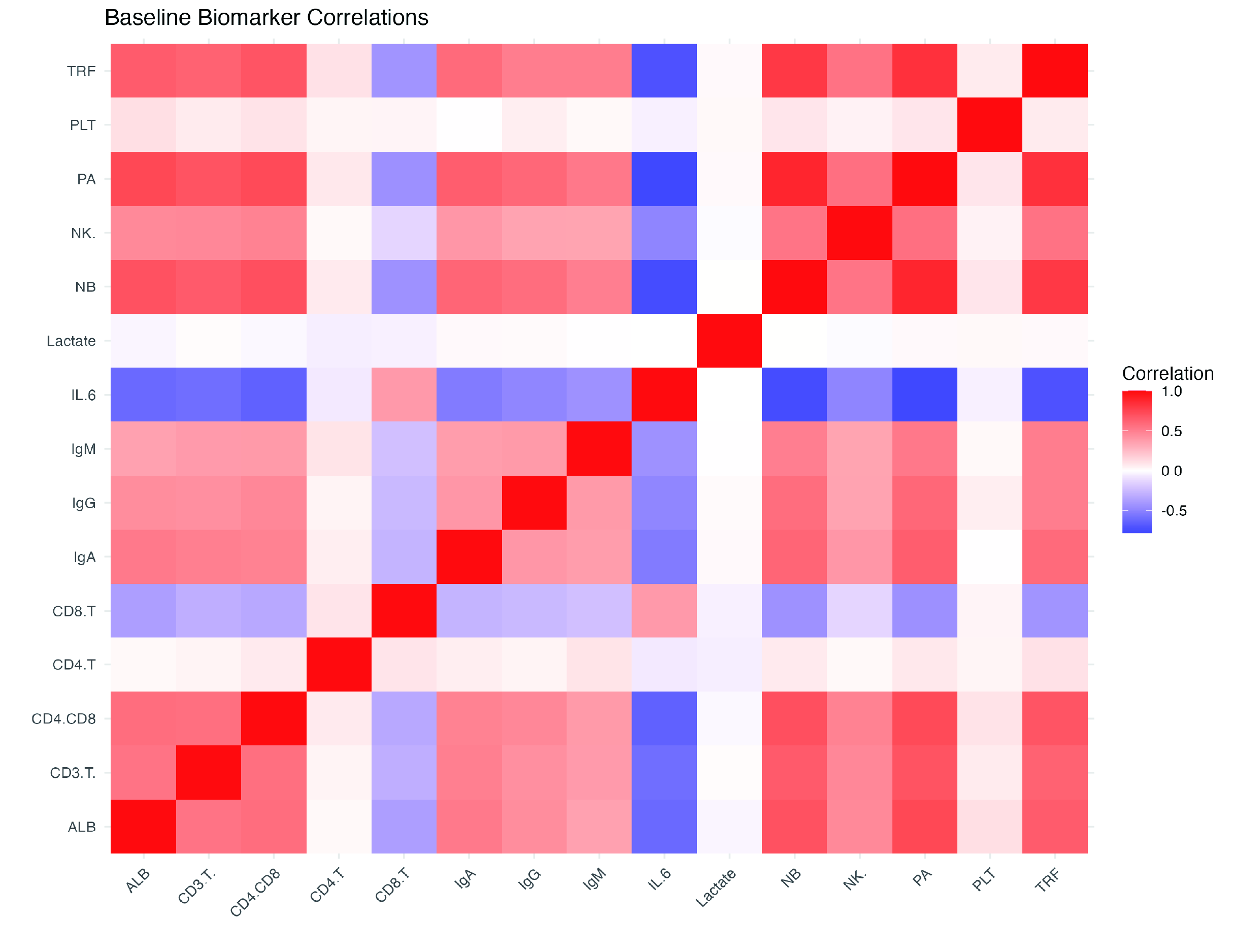

Supplement: Supplementary Figure 2 — Pearson Correlation Matrix of Day 1 Biomarker Levels. Heatmap displaying pairwise Pearson correlation coefficients among all 15 biomarkers measured at baseline (Day 1). The color scale ranges from blue (negative correlation) through white (no correlation) to red (positive correlation). Correlations were calculated using pairwise complete observations. This matrix illustrates the interrelationships among nutritional, immunological, and inflammatory markers, informing the multicollinearity observed in multivariable models. [file Image2.tif]
