# Supplementary material for: Dynamic biomarker profiling and phenotyping in burn sepsis: a retrospective cohort study using growth mixture modeling
Source: Front Cell Infect Microbiol. 2026 Apr 16;16:1710916. doi: 10.3389/fcimb.2026.1710916 (PMC13128616; doi:10.3389/fcimb.2026.1710916)
Supplement: Supplementary file 3 [file Table1.docx]

| **Supplementary Table S1. Variance Inflation Factors for Variables in the Multivariable Cox Regression Model** | |
| --- | --- |
| **Variable** | **Variance Inflation Factor (VIF)** |
| Age | 1.29 |
| NRS2002 | 1.55 |
| Burn Index | 3.89 |
| NK cells_1d | 1.56 |
| TRF_1d | 4.23 |
| CD4/CD8 ratio_1d | 2.46 |
| NB_1d | 5.71 |
| PA_1d | 8.42 |
| *VIF values > 5 indicate substantial multicollinearity. Abbreviations: NK, natural killer; TRF, transferrin; NB, nitrogen balance; PA, prealbumin.* | |
