# Supplementary material for: Dynamic biomarker profiling and phenotyping in burn sepsis: a retrospective cohort study using growth mixture modeling
Source: Front Cell Infect Microbiol. 2026 Apr 16;16:1710916. doi: 10.3389/fcimb.2026.1710916 (PMC13128616; doi:10.3389/fcimb.2026.1710916)
Supplement: Supplementary file 4 [file Table2.docx]

| **Supplementary Table S2. Longitudinal Profiles of Biomarkers Stratified by 21-Day Survival Status** | | | |
| --- | --- | --- | --- |
| **Biomarker** | **Time point** | **Survivors (n=631) Mean ± SD** | **Non‑survivors (n=81) Mean ± SD** |
| ALB (g/L) | Day 1 | 36.29 ± 10.52 | 33.43 ± 11.01 |
|  | Day 3 | 32.59 ± 8.11 | 30.51 ± 9.30 |
|  | Day 7 | 31.52 ± 5.74 | 31.25 ± 5.37 |
|  | Day 14 | 33.98 ± 4.92 | 34.07 ± 5.12 |
|  | Day 21 | 35.91 ± 4.55 | 35.32 ± 5.46 |
| PA (mg/L) | Day 1 | 239.22 ± 111.95 | 203.33 ± 120.54 |
|  | Day 3 | 213.04 ± 106.50 | 178.16 ± 110.49 |
|  | Day 7 | 220.27 ± 67.87 | 202.93 ± 71.50 |
|  | Day 14 | 243.92 ± 66.34 | 230.57 ± 72.55 |
|  | Day 21 | 262.21 ± 55.22 | 243.99 ± 58.84 |
| TRF (g/L) | Day 1 | 166.11 ± 39.46 | 150.87 ± 39.23 |
|  | Day 3 | 149.51 ± 35.37 | 138.07 ± 34.18 |
|  | Day 7 | 167.91 ± 48.93 | 153.41 ± 52.52 |
|  | Day 14 | 178.04 ± 41.82 | 165.65 ± 47.85 |
|  | Day 21 | 214.06 ± 11.91 | 211.77 ± 15.92 |
| NB (g/24h) | Day 1 | 0.13 ± 1.51 | -0.35 ± 1.64 |
|  | Day 3 | 0.23 ± 1.56 | -0.32 ± 1.70 |
|  | Day 7 | 0.57 ± 1.07 | 0.20 ± 1.13 |
|  | Day 14 | 1.27 ± 0.46 | 1.16 ± 0.49 |
|  | Day 21 | 1.40 ± 0.71 | 1.35 ± 0.70 |
| IgA (g/L) | Day 1 | 2.83 ± 1.46 | 2.57 ± 1.49 |
|  | Day 3 | 1.93 ± 1.17 | 1.74 ± 1.12 |
|  | Day 7 | 2.02 ± 1.29 | 1.86 ± 1.28 |
|  | Day 14 | 2.29 ± 1.40 | 2.04 ± 1.19 |
|  | Day 21 | 2.70 ± 1.23 | 2.63 ± 1.31 |
| IgG (g/L) | Day 1 | 13.65 ± 5.96 | 11.95 ± 5.18 |
|  | Day 3 | 9.53 ± 3.37 | 8.69 ± 3.20 |
|  | Day 7 | 9.97 ± 4.32 | 8.89 ± 3.50 |
|  | Day 14 | 12.45 ± 4.24 | 11.88 ± 3.39 |
|  | Day 21 | 14.29 ± 4.35 | 14.15 ± 4.32 |
| IgM (g/L) | Day 1 | 1.43 ± 0.32 | 1.35 ± 0.26 |
|  | Day 3 | 1.17 ± 0.32 | 1.16 ± 0.28 |
|  | Day 7 | 1.12 ± 0.22 | 1.11 ± 0.25 |
|  | Day 14 | 1.29 ± 0.45 | 1.18 ± 0.37 |
|  | Day 21 | 1.45 ± 0.31 | 1.47 ± 0.39 |
| CD3⁺ T cells (%) | Day 1 | 61.45 ± 9.75 | 58.79 ± 11.62 |
|  | Day 3 | 54.66 ± 9.55 | 52.06 ± 10.31 |
|  | Day 7 | 52.84 ± 9.23 | 52.07 ± 10.13 |
|  | Day 14 | 56.02 ± 10.84 | 55.74 ± 13.53 |
|  | Day 21 | 63.06 ± 6.97 | 60.95 ± 6.90 |
| CD4⁺ T cells (%) | Day 1 | 10.17 ± 5.08 | 8.93 ± 4.90 |
|  | Day 3 | 8.52 ± 3.39 | 9.11 ± 3.04 |
|  | Day 7 | 8.78 ± 2.90 | 8.94 ± 2.95 |
|  | Day 14 | 14.07 ± 3.88 | 14.14 ± 3.55 |
|  | Day 21 | 15.83 ± 4.14 | 16.05 ± 4.34 |
| CD8⁺ T cells (%) | Day 1 | 12.06 ± 4.17 | 13.05 ± 4.39 |
|  | Day 3 | 14.11 ± 4.13 | 14.58 ± 4.48 |
|  | Day 7 | 16.33 ± 4.68 | 16.53 ± 4.85 |
|  | Day 14 | 13.27 ± 4.30 | 13.44 ± 5.32 |
|  | Day 21 | 10.59 ± 3.42 | 10.46 ± 3.84 |
| CD4⁺/CD8⁺ ratio | Day 1 | 1.28 ± 0.40 | 1.14 ± 0.36 |
|  | Day 3 | 0.95 ± 0.38 | 0.83 ± 0.37 |
|  | Day 7 | 0.66 ± 0.32 | 0.60 ± 0.16 |
|  | Day 14 | 1.03 ± 0.34 | 1.06 ± 0.36 |
|  | Day 21 | 1.49 ± 0.41 | 1.41 ± 0.53 |
| NK cells (%) | Day 1 | 20.32 ± 7.44 | 17.41 ± 7.17 |
|  | Day 3 | 16.43 ± 5.80 | 15.56 ± 5.41 |
|  | Day 7 | 12.81 ± 5.94 | 12.26 ± 7.04 |
|  | Day 14 | 21.62 ± 7.69 | 21.09 ± 6.07 |
|  | Day 21 | 32.17 ± 8.00 | 30.48 ± 8.09 |
| IL‑6 (pg/mL) | Day 1 | 184.26 ± 67.26 | 205.77 ± 67.43 |
|  | Day 3 | 177.61 ± 69.78 | 201.26 ± 73.95 |
|  | Day 7 | 156.06 ± 48.86 | 172.91 ± 44.60 |
|  | Day 14 | 122.04 ± 46.81 | 137.29 ± 47.66 |
|  | Day 21 | 103.75 ± 43.62 | 118.04 ± 53.47 |
| PLT (×10⁹/L) | Day 1 | 214.81 ± 20.97 | 211.94 ± 20.61 |
|  | Day 3 | 138.25 ± 34.90 | 142.07 ± 34.54 |
|  | Day 7 | 106.54 ± 26.74 | 102.10 ± 27.34 |
|  | Day 14 | 188.95 ± 64.04 | 184.80 ± 63.68 |
|  | Day 21 | 224.43 ± 42.59 | 225.58 ± 41.94 |
| Lactate (mmol/L) | Day 1 | 3.25 ± 0.43 | 3.23 ± 0.44 |
|  | Day 3 | 1.99 ± 0.29 | 1.96 ± 0.30 |
|  | Day 7 | 1.48 ± 0.29 | 1.47 ± 0.27 |
|  | Day 14 | 1.30 ± 0.29 | 1.28 ± 0.27 |
|  | Day 21 | 1.00 ± 0.29 | 1.03 ± 0.30 |
| *Abbreviations: ALB, albumin; PA, prealbumin; TRF, transferrin; NB, nitrogen balance; Ig, immunoglobulin; NK, natural killer; IL‑6, interleukin‑6; PLT, platelet count; SD, standard deviation.* | | | |
